# Sharps injuries and bloodborne pathogen exposure among healthcare workers: a 5-year surveillance in a teaching hospital in Singapore

**DOI:** 10.1017/ash.2025.105

**Published:** 2025-09-03

**Authors:** Zhang Zhimin, Jin Pinhong, Toh Hui Xian, Tan Soong Geck, Molly How Kue Bien, Tan Kwee Yuen, Sheena Ong Jin Min, Lee Lai Chee, Ling Moi Lin

**Affiliations:** Infection Prevention & Epidemiology, Singapore General Hospital

## Abstract

**Introduction:** Bloodborne pathogens’ exposures are defined as injuries to contaminated sharps and exposures to patients’ blood or body fluids continue to present risks to healthcare workers (HCWs). This study was conducted to investigate the epidemiological characteristics of sharps injuries and evaluate the effectiveness of prevention strategies in an Academic Medical Centre (AMC) in Singapore. **Method:** This retrospective study was conducted at a 1,700 bedded AMC. The data was retrieved from the hospital’s electronic incident reporting system over a five-year period between 2019 and 2023 at Singapore General Hospital. **Results:** There are a total of 719 bloodborne pathogens’ exposures incidents. The highest incidence of bloodborne pathogens’ exposures was reported among doctors (3.5 incidents per 1000 healthcare workers per month), followed by nurses (1.4), allied health (0.4) and ancillary staff (0.3) during the 5- year period. Intraoperative procedures (IOP) 198 (27.5%) see the most frequent incidents followed by minor procedures 112(15.6%), splash incidents 91 (12.7%), blood taking 87(12.1%), IVIM (8.3%). The highest incidents among IOP were associated with use of suture needles. Approximately 2.9%, 2.3% and 0.7% of the source patients were carriers for Hepatitis B, Hepatitis C and HIV respectively. No seroconversion occurred among all injured HCWs. The overall sharps injury incidence has improved from 21.5 to 11.2 per 1000 healthcare workers per year following targeted preventive measures implementation. **Conclusion:** A comprehensive bloodborne pathogen exposures programme greatly helps to improve and mitigate the risk of exposures where key preventive measures are identified, followed by timely implementation of appropriate post-exposure management.

